# Supplementary figures and images for: Human cardiac fibroblasts adaptive responses to controlled combined mechanical strain and oxygen changes in vitro
Source: eLife. 2017 Mar 18;6:e22847. doi: 10.7554/eLife.22847 (PMC5407858; doi:10.7554/eLife.22847)

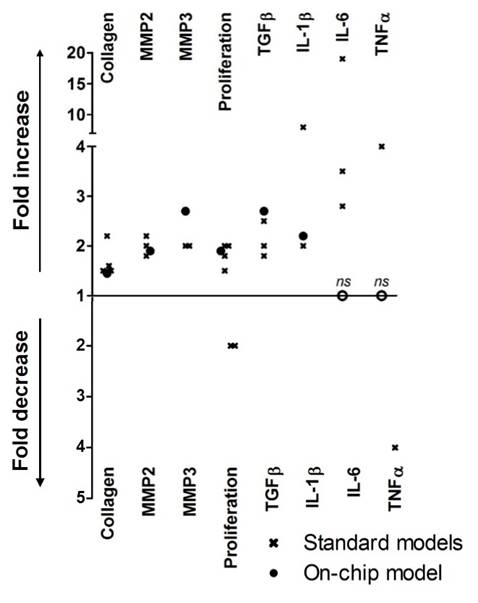

Supplement: Table 2—source data 1. — DOI: http://dx.doi.org/10.7554/eLife.22847.014 [file elife-22847-table2-data1.jpg]
